# An Experiment Certified

**Published:** 1855-12

**Authors:** 


### AN EXPERIMENT CERTIFIED.

*The* Dr. Jackson of Boston, in his dedication of *Letters to a Young Physician*, to *the* Dr. Warren of the same city, states that, half a century ago, they began an experiment which has now terminated.

It is a coincidence worthy of note, that two other men in the mercantile profession, began a similar experiment in the same city. The merchants completed their experiments with their lives, while the two doctors *still live* to contemplate the result of their own. Both these experiments were successful; not moderately successful, but splendidly so. Successful, not merely in the accumulation of money, but what has infinitely more than money's worth, in attaining high social position, and a character for integrity, spotless; and more still, in a deserved reputation for Philanthropy, whose lustre dims the coronets of kings.

Amos and Abbott Lawrence began life poor: they determined that the strictest integrity should pervade every business transaction until their dying hour, and it was so. Among the results are the accumulation of millions of money, the possession of a name for mercantile integrity, worth more to them, tc their children, to their age and nation, than a title to a dukedom; while they did, during life, and at death, institute charities, which will heap sweet blessings on their name and memory for ages yet to come. Let every merchant's clerk on this broad earth make that same experiment, and take encouragement from the assurance, founded in the very nature of things, that similar results will accrue to him.

The experiment of the two eminent physicians we have named, is equally worthy the practice of every young gentleman who aspires to the honorable and responsible position of doctor of medicine. Away with all schools here, let them sink for the present, to some bottomless abyss. It was not an experiment *for* allopathy, (but we do take pride in saying it was made *by* allopathy !) nor for eclecticism or cold water, not for the herbiverous or infinitessimal schools; it was made for the educated and honorable practitioners of all schools. And what was proven by it? That two rival physicians may live in the same town for more than fifty years, and during that long period of almost daily intercourse not originate one disparaging word or act, exhibit no uncourteous feeling, throw out no depreciating inuendo, commit no breach of professional etiquette; in short, do no deed which, if communicated, need irritate the one or cause a blush to mantle the cheek of the other.

On the contrary, they were always ready to help each other and to promote each other's happiness. Not resembling in temperament, they often differed in opinion, but always agreed to differ; sometimes disputed, but never quarrelled. Each gave the other the credit due him, neither trying to push the other aside, and, in the front rank of their profession, both continue on terms of intimacy and friendship to this day.

How grandly elevating must be the retrospection of Jackson and Warren! What a perennial spring of sweet memories well up in their hearts, as often as they turn their eyes inwards and backwards on so long and honorable and successful a career! and beside them, how sink they to depths which no plummet can reach, those poor unfortunates found in the village as well as in the city, not only in this but in other callings, who seek to elevate themselves at the expense of their rivals! too often ending in mutual ruin, while, if on the other hand, equal pains were taken to sustain each other, the ordinary result would be similar to the one we have chronicled. And it is very pertinent to add here, that but for an *injudicious change in dress,* Abbott Lawrence might still be living in the enjoyment of his success.

